# Assessment of Validity of a Blood-Based 3-Gene Signature Score for Progression and Diagnosis of Tuberculosis, Disease Severity, and Treatment Response

**DOI:** 10.1001/jamanetworkopen.2018.3779

**Published:** 2018-10-26

**Authors:** Hayley C. Warsinske, Aditya M. Rao, Flora M. F. Moreira, Paulo Cesar P. Santos, Andrew B. Liu, Madeleine Scott, Stephaus T. Malherbe, Katharina Ronacher, Gerhard Walzl, Jill Winter, Timothy E. Sweeney, Julio Croda, Jason R. Andrews, Purvesh Khatri

**Affiliations:** 1 Institute for Immunity, Transplantation, and Infection, Stanford University, Stanford, California; 2 Center for Biomedical Informatics, Department of Medicine, Stanford University, Stanford, California; 3 Stanford Immunology Graduate Program, Stanford University, Stanford, California; 4 Federal University of Grande Dourados, Dourados, Brazil; 5 Stanford Biophysics Graduate Program, Stanford University, Stanford, California; 6 South African Medical Research Council Centre for Tuberculosis Research, Division of Molecular Biology and Human Genetics, Faculty of Medicine and Health Sciences, Stellenbosch University, Cape Town, South Africa; 7 Centre of Excellence for Biomedical Tuberculosis Research, Department of Science and Technology-National Research Foundation, Stellenbosch University, Cape Town, South Africa; 8 Catalysis Foundation for Healthy, Emeryville, California; 9 Federal University of Mato Grosso do Sul, Campo Grande, Brazil; 10 Oswaldo Cruz Foundation, Mato Grosso Sul, Campo Grande, Brazil; 11 Division of Infectious Diseases and Geographic Medicine, Department of Medicine, Stanford University, Stanford, California

## Abstract

**Importance:**

The World Health Organization identified the need for a non–sputum-based triage test to identify those in need of further tuberculosis (TB) testing.

**Objective:**

To determine whether the 3-gene TB score can be a diagnostic tool throughout the course of TB disease, from latency to diagnosis to treatment response, and posttreatment residual inflammation.

**Design, Setting, and Participants:**

This nested case-control study analyzed the 3-gene TB score in 3 cohorts, each focusing on a different stage of TB disease: (1) the Adolescent Cohort Study profiled whole-blood samples from adolescents with latent *Mycobacterium tuberculosis* infection, some of which progressed to active TB (ATB), using RNA sequencing; (2) the Brazil Active Screen Study collected whole blood from an actively screened case-control cohort of adult inmates from 2 prisons in Mato Grosso do Sul, Brazil, for ATB from January 2016 to February 2016; and (3) the Catalysis Treatment Response Cohort (CTRC) identified culture-positive adults in primary health care clinics in Cape Town, South Africa, from 2005 to 2007 and collected whole blood for RNA sequencing from patients with ATB at diagnosis and weeks 1, 4, and 24. The CTRC patients also had positron emission tomography–computed tomography scans at diagnosis, week 4, and week 24. Analyses were performed from September 2017 to June 2018.

**Main Outcomes and Measures:**

A 3-gene messenger RNA expression score, measured by quantitative polymerase chain reaction or RNA sequencing, was evaluated for distinguishing the following: individuals who progressed to ATB from those who did not, individuals with ATB from those without, and individuals with slower treatment response during TB therapy.

**Results:**

Patients evaluated in this study included 144 adolescents from the Adolescent Cohort Study (aged 12-18 years; 96 female and 48 male), 81 adult prison inmates from the Brazil Active Screen Study (aged 20-72 years; 81 male), and 138 adult community members from the CTRC (aged 17-64 years; 81 female and 57 male). The 3-gene TB score identified progression from latent *M tuberculosis* infection to ATB 6 months prior to sputum conversion with 86% sensitivity and 84% specificity (area under the curve [AUC], 0.86; 95% CI, 0.77-0.96) and patients with ATB in the Brazil Active Screen Study cohort (AUC, 0.87; 95% CI, 0.78-0.95) and CTRC (AUC, 0.94; 95% CI, 0.88-0.99). It also identified CTRC patients with failed treatment at the end of treatment (AUC, 0.93; 95% CI, 0.83-1.00). Collectively, across all cohorts, the 3-gene TB score identified patients with ATB with 90% sensitivity, 70% specificity, and 99.3% negative predictive value at 4% prevalence.

**Conclusions and Relevance:**

Across 3 independent prospective cohorts, the 3-gene TB score approaches the World Health Organization target product profile benchmarks for non–sputum-based triage test with high negative predictive value. This gene expression diagnostic approach should be considered for further validation and future implementation.

## Key Points

**Question:**

How does a previously described blood-based 3-gene tuberculosis score perform as a screening test and a disease monitoring tool for all stages of tuberculosis?

**Findings:**

In this nested case-contral study, the 3-gene tuberculosis score was associated with progression from latent *Mycobacterium tuberculosis* infection to active tuberculosis 6 months prior to sputum conversion with 86% sensitivity and 84% specificity, diagnosed patients with active tuberculosis with 90% sensitivity and 70% specificity, and correlated with treatment response and the severity of lung pathology.

**Meaning:**

The 3-gene tuberculosis score can be implemented as a rapid, blood-based screening and triage test with the required World Health Organization target product profiles for the accurate detection and tracking of progressive, active, and treated tuberculosis disease.

## Teaser

This nested case-control study analyzes 3 independent cohorts to determine whether the 3-gene tuberculosis (TB) score can be a diagnostic tool throughout the course of TB disease and posttreatment residual inflammation by distinguishing individuals who progress to active TB vs those who do not, those with vs without active TB, and those with slower treatment response.

## Introduction

As part of its End Tuberculosis (TB) 2035 strategy to reduce the prevalence and burden of TB, the World Health Organization (WHO) consensus meeting report of 2014 has asked for a triage test to rule out disease and for systematic screening that can be used by first-contact clinicians to identify patients who need further testing with 90% sensitivity and 70% specificity.^1^ A rule-out triage test should have high negative predictive value (NPV) to have high confidence that the individual with a negative test result indeed does not need to be subjected to additional tests.^1^ The WHO has also published the target product profile (TPP) for a test anticipating progression from latent *Mycobacterium tuberculosis* infection (LTBI) to active TB (ATB) disease with specificity greater than 75% and sensitivity greater than 75%.^2^ In addition, the Foundation for Innovative New Diagnostics (FIND) and the New Diagnostics Working Group of the Stop TB Partnership have drafted an intervention TPP for a new prognostic test for TB risk^3^ that proposes a new test would need to increase the positive predictive value (PPV) to at least 5.8%.

There are currently no commercially available tests, to our knowledge, that satisfy these criteria. Solid culture results take weeks to provide a diagnosis and liquid culture grown in the mycobacteria growth indicator tube (MGIT) still requires 7 to 10 days for a positive diagnosis and 42 days before negativity can be declared. Sputum-smear microscopy is the most commonly used ATB diagnostic, but has only 45% to 61% sensitivity^4,5,6,7^ and cannot detect ATB before patients become infectious. The tuberculin skin test cannot differentiate between ATB and LTBI.^8,9^ Interferon gamma release assay has limited value in anticipating progression from latency to active disease.^9,10,11^ GeneXpert MTB/RIF has improved on previous standards,^12^ but is limited by dependence on sputum.

Several host response-based transcriptional signatures have been described for diagnosis of ATB^13,14,15,16,17,18,19,20^ and for anticipating progression from latent to active disease.^21^ However, most of these signatures are not suitable for translation to clinical practice because of the following: (1) dependence on a large number of genes inhibiting development of a cost-effective assay,^13,19,20^ (2) lack of extensive validation in diverse and independent cohorts that represent real-world heterogeneity of patients with TB, as well as validation in independent technological platforms that are appropriate for point-of-care diagnostics, (3) confounding by age or coinfection with HIV,^13,19,20^ and (4) lack of specificity for ATB compared with other lung diseases. Further, none of these host response-based signatures have been shown to be associated with the lung pathology during ATB to date.

We previously described a 3-gene signature derived and validated in a multicohort analysis of 2572 whole-blood transcriptome profiles in 14 independent cohorts from 11 countries that addresses many of these challenges.^18^ A score derived from this 3-gene signature distinguished patients with ATB from those with other diseases, LTBI, and healthy controls, irrespective of age, HIV coinfection, and prior bacille Calmette-Guérin (BCG) vaccination.^18^ Importantly, the 3-gene TB score was identified and retrospectively validated using clinical samples from multiple countries, demonstrating its robustness to differences in genetic background of patients and *M tuberculosis* (*Mtb*) strains, and using microarrays from 4 manufacturers, showing robustness to different messenger RNA profiling technologies. Across these studies, the 3-gene TB score had 99% NPV at 10% prevalence.

In this article, we prospectively assessed the validity of the 3-gene TB score in 3 independent cohorts for association with progression from LTBI to ATB in the Adolescent Cohort Study (ACS) from South Africa, diagnosis of ATB in the Brazil Active Screen Study (BASS), and association with ATB severity and treatment response in the Catalysis Treatment Response Cohort (CTRC) from South Africa.

## Methods

### 3-Gene TB Score

The 3-gene TB score in microarray and RNA sequencing (RNA-seq) data sets is calculated as^18^

3-gene TB score = ([log(*GBP5*) + log(*DUSP3*)]/2) − log(*KLF2*)

where log(*GBP5*), log(*DUSP3*), and log(*KLF2*) are normalized log_2_-transformed mean fluorescence intensity or normalized read count values of *GBP5*, *DUSP3*, and *KLF2*.

For the BASS cohort the 3-gene TB score using quantitative reverse transcription–polymerase chain reaction (RT-qPCR) is defined as

3-gene TB score = ([(*GBP5*) + (*DUSP3*)]/2) − *KLF2*

where delta Ct values from RT-qPCR for each gene in each sample are normalized to a housekeeping gene (*POLG1*) and used in the above equation.

The 3-gene TB score was calculated for every sample described in this article and used for all statistical comparisons relevant to that sample.

### Cohort Descriptions

#### The Adolescent Cohort Study

The ACS cohort has been previously described by other groups.^17,21,22^ Briefly, in the ACS, adolescents with LTBI were enrolled in a rural community in Western Cape, South Africa from 2005 to 2007.^17^ Whole-blood samples from 153 LTBI adolescents were profiled using RNA-seq. However, phenotypic information was only available for 144 individuals. Therefore, 9 adolescents were excluded from further analysis. Of the 144 individuals, 43 developed ATB during the study (called progressors); the remaining 101 individuals are referred to as nonprogressors. In this cohort, we investigated whether the 3-gene TB score could identify those progressing from LTBI to ATB significantly earlier than the sputum-based diagnosis of ATB (eAppendix in the Supplement). The study protocols were approved by the University of Cape Town Research Ethic Committee Cape Town, South Africa. Written informed consent was obtained from participants. For adolescents, consent was obtained from parents or legal guardians of adolescents and written informed assent from each adolescent.

#### Brazil Active Screening Study Cohort

The BASS is a nested case-control study that prospectively enrolled inmates from Estabelecimento Penal Jair Ferreira de Carvalho and Dourados State Prison in Campo Grande and Dourados, Brazil, respectively. The study was approved by the research ethics committee at the Federal University of Grande Dourados, National Commission on Ethics in Research (CAAE: 44997115.1.0000.5160), and Stanford University institutional review board. Every patient with ATB identified during the study was notified and underwent treatment. Prisoners provided written informed consent in a private room without involvement of prison staff. Participation decisions were not reported to prison staff unless an individual was diagnosed with TB, in which case the diagnosis was reported, as mandated by Brazilian law, and free treatment was provided. We followed the Strengthening the Reporting of Observational Studies in Epidemiology (STROBE) reporting guideline.

Between January 2016 and February 2016, all 3680 inmates aged 18 years or older at these 2 prisons were recruited, of whom 105 declined consent; the remaining 3575 inmates consented to participate and were enrolled. Individuals were not excluded from the study for any potential comorbidities. Participants demographic (eTable 1 in the Supplement) and clinical information was ascertained by a standard questionnaire (eAppendix in the Supplement).

All participants reporting a cough or any other WHO-defined TB symptoms were asked to provide sputum for an assessment of ATB. Two sputum samples were collected, including 1 spot sample after the interview and another the next morning, consistent with the WHO recommendations at the time. Smear microscopy (Ziehl-Neelsen) and solid culture were used to test for *Mtb*. Samples were decontaminated using the Petroff method and culturing was performed using modified Ogawa medium^23,24,25^ and maintained for 60 days until it was considered negative. Solid media was used because MGIT was not available at the state TB laboratory where the study was performed. Radiography was not available in the prisons, and a TB case was therefore defined as the presence of at least 1 positive culture test.

In these prisons, previous studies found that 1.5% of inmates had HIV infection^26,27^; we did not exclude HIV-infected individuals from the study, but happened to have none among the 81 participants. Their demographic (eTable 1 in the Supplement) and clinical information was ascertained by a standard questionnaire, and 2 sputum samples and whole-blood samples in PAXgene RNA tubes were collected.

All ATB cases, defined as a positive sputum culture for *Mtb,* and consecutive controls that were sputum culture negative were included in gene expression analysis on a case to control ratio of 1 to 1.5.

#### The CTRC Cohort

The CTRC has been previously described by other groups.^21,22,28^ In the CTRC, whole-blood samples from 138 HIV-negative adults (aged 17-67 years), who were enrolled in primary health care clinics in Cape Town, South Africa, were profiled using RNA-seq from diagnosis and prior to treatment initiation until the end of their treatment. These 138 adults include 100 MGIT culture-positive patients with ATB, 21 healthy controls, and 17 patients with other lung diseases (pneumonia or asthma). Patients with ATB received standard care of 2 months (isoniazid, rifampin, pyrazinamide, and ethambutol)/4 months (isoniazid and rifampin)^29^ treatment following diagnosis. Biometrics were collected for these patients at the time of diagnosis prior to treatment initiation (baseline), 1 week, 4 weeks, and 24 weeks (study-defined end of treatment [EOT] for all patients) (eAppendix in the Supplement). Biometrics collected included, but were not limited to, positron emission tomography–computed tomography (PET-CT) scores, MGIT culture tests, and whole-blood gene expression using RNA-seq. The complete list of biometrics has been previously described.^22^ The PET-CT images were evaluated in collaboration and consensus with a radiologist, a nuclear physician, and a pulmonologist^22^ at Stellenbosch University in association with the Tygerburg PET/CT facility. Patient clinical outcomes were not known at the time of reading.

Sputum cultures were performed throughout the course of the study. Patients who completed therapy and had at least the last 2 consecutive sputum cultures at the EOT as negative were considered cured. Seven patients with positive sputum test at the EOT were considered to have failed treatment. Two patients with EOT contaminated sputum cultures were considered unevaluable and were removed from the analysis. Ethical approval was obtained from the Stellenbosch University Human Research Ethics Committee. Written informed consent was obtained from participants.

### Outcomes

For the ACS,^17^ our primary outcome was diagnosis of ATB, and the secondary outcome was association with progression from latent to active disease.^30^ Our primary outcome in the BASS was diagnosis of ATB. For the CTRC,^21,22,28^ our primary outcome was correlation between 3-gene TB score and lung pathology measured as Total Glycolytic Activity Index (TGAI). Secondary outcomes for the CTRC included the association of persistent TGAI at the EOT with baseline 3-gene TB score and a hazard ratio of prolonged lung pathology if the baseline 3-gene TB score is above the median 3-gene TB score for patients with ATB.

### Statistical Analysis

For each data set we report sensitivity closest to 90% (as specified in the WHO TPP) and the corresponding specificity. Overall sensitivity and specificity values for diagnosis of ATB across the 3 cohorts were calculated by pooling true-positives, false-positives, true-negatives, and false-negatives from the 3 data sets. The pooled values were then used to calculate sensitivity and specificity to give total sensitivity and total specificity. We computed PPV and NPV according to the methods described by Altman and Bland,^31^ and estimated according to Bayes theorem,^32^ assuming 4% prevalence (eAppendix in the Supplement).

## Results

We evaluated the performance of the 3-gene TB score in 3 prospective cohorts of patients with ATB (eTable 2 in the Supplement).

### Diagnosis of ATB by 3-Gene TB Score 6 Months Prior to Positive Sputum Test in the ACS

**Figure 1.**
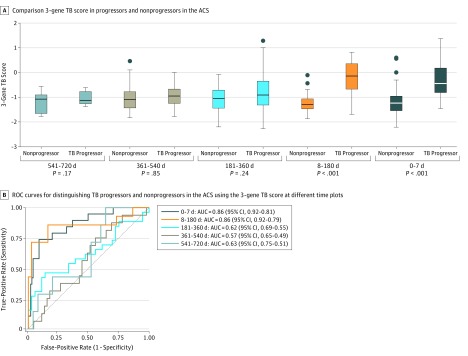
Association of the 3-Gene TB Score With Progression From Latent to Active Disease 6 Months Prior to Sputum Conversion in the ACS A, Box plots comparing the distributions of 3-gene tuberculosis (TB) scores between progressors and nonprogressors collected at 720 to 541, 540 to 361, 360 to 181, 180 to 8, and 7 to 0 days prior to sputum conversion. The horizontal line in the middle of each box indicates the median, while the bottom and top borders of the box represent the first and third quartile, respectively. The whiskers above and below represent the range of values. Circles indicate outliers. B, Receiver operating characteristics (ROC) curves for distinguishing progressors and nonprogressors prior to sputum conversion at different intervals. ACS indicates Adolescent Cohort Study; AUC, area under the curve.

Among 144 adolescents with LTBI in the ACS, 43 progressed to ATB (progressors) and the rest remained LTBI (nonprogressors). A linear mixed-effects regression analysis found a significant effect of the interaction between time and progressor status (*P* = .006; Figure 1A) but no significant effect of time alone (*P* = .17; Figure 1A).^33^ The 3-gene TB score was significantly higher in progressors compared with nonprogressors within 7 days of diagnosis (*P* < .001; Figure 1A). At 89.47% sensitivity, the 3-gene TB score achieved 63.37% specificity and 99.13% NPV at 4% prevalence (area under receiver operator curve [AUROC], 0.86; 95% CI, 0.77-0.96) (Figure 1B and Table) within 7 days of diagnosis.

**Table.** Sensitivity, Specificity, and NPV of 3-Gene Tuberculosis Score as a Triage Test for Active Tuberculosis and a Test for Progression From Latent *Mycobacterium Tuberculosis* Infection to Active Tuberculosis

Cohort	%			AUROC (95% CI)
	Sensitivity	Specificity	NPV at 4% Prevalence	
WHO test name				
Triage test				
BASS	90.91	68.75	99.29	0.87 (0.79-0.94)
ACS, 0-7 d	89.47	63.37	99.13	0.86 (0.77-0.96)
CTRC	90.11	89.19	99.42	0.94 (0.88-0.99)
Overall	90	70	99.3	
Progression test				
ACS, 8-180 d	86	84	98.63	0.86 (0.70-1.00)

Abbreviations: ACS, Adolescent Cohort Study; AUROC, area under receiver operating characteristic curve; BASS, Brazil Active Screen Study; CTRC, Catalysis Treatment Response Cohort; NPV, negative predictive value; WHO, World Health Organization.

Further, the 3-gene TB score was higher in the progressors compared with nonprogressors when using only the samples obtained between 8 days to 180 days prior to positive sputum microscopy (*P* < .001; Figure 1A). At 86% sensitivity, in this time window, it distinguished progressors from nonprogressors with 84% specificity and 98.63% NPV at 4% prevalence (AUROC, 0.86; 95% CI,0.70-1.00) (Figure 1B and Table). The 3-gene TB score did not distinguish progressors from nonprogressors in samples collected more than 6 months prior (Figure 1).

### Active Case Detection of ATB by 3-Gene TB Score in the BASS

**Figure 2.**
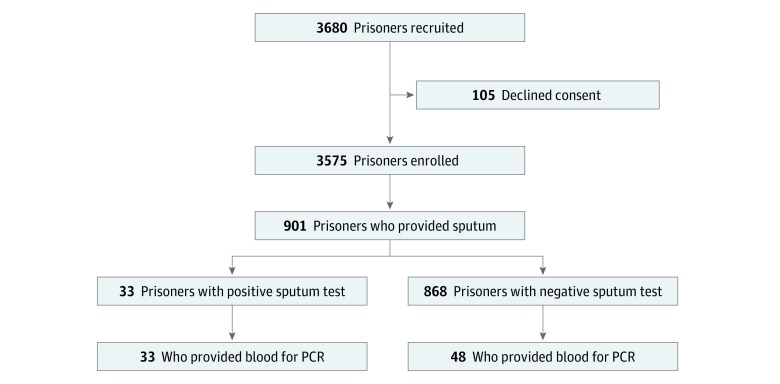
Flowchart of the Screening Process in the Brazil Active Screen Study Cohort for the Detection of Active Tuberculosis PCR indicates polymerase chain reaction.

**Figure 3.**
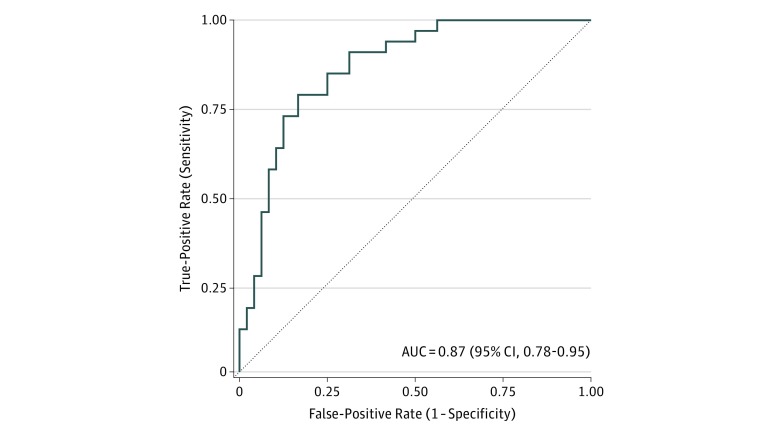
Diagnosis of Patients With Active Tuberculosis With High Accuracy in Active Screening in the Brazil Active Screen Study Cohort Solid line indicates receiver operating characteristic curve distinguishing patients with active tuberculosis from patients without tuberculosis in the Brazil Active Screen Study cohort. AUC indicates area under the curve.

For active case detection in the BASS (Figure 2), we prospectively recruited all 3680 inmates from Estabelecimento Penal Jair Ferreira de Carvalho and Dourados State Prison in Campo Grande and Dourados, Brazil, respectively, of whom 3575 inmates (97%) consented to screening. Of 901 (25%) who provided sputum, we found 33 cases of ATB for a point prevalence of 920 per 100 000 prisoners (95% CI, 660-1290). We measured expression of the 3 genes in the TB signature using RT-qPCR in all 33 ATB cases and 48 consecutive controls (Figure 2 and eTable 1 in the Supplement). At 90.91% sensitivity, the 3-gene TB score achieved 68.75% specificity and 99.29% NPV at 4% prevalence for actively screened cases (AUROC, 0.87; 95% CI, 0.79-0.94) (Figure 3).

### Correlation Between 3-Gene TB Score and Lung Pathology and Treatment Response in the CTRC Cohort

The CTRC profiled 138 HIV-negative individuals (100 patients with ATB, 21 healthy controls, and 17 patients with pneumonia or asthma) using RNA-seq.^22^ Of 100 patients with ATB, samples from 2 patients were contaminated and removed from further analysis. Of the remaining 98 patients with ATB, 7 failed treatment.

We compared the 3-gene TB score of the patients with ATB with that of healthy controls and patients with other lung diseases at the time of diagnosis. At 90.11% sensitivity, the 3-gene TB score achieved 89.19% specificity and 99.42% NPV at 4% prevalence for distinguishing patients with ATB from healthy controls and patients with other lung diseases (AUROC, 0.94; 95% CI, 0.88-0.99) (eFigure 1 in the Supplement). Further, the 3-gene TB score of the patients with ATB at the EOT (day 168) also distinguished patients who had failed treatment (AUROC, 0.93; 95% CI, 0.83-1.00) (eFigure 1 in the Supplement).

**Figure 4.**
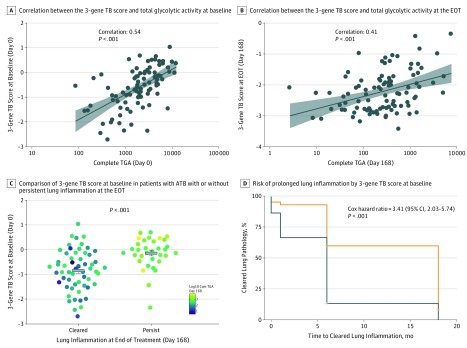
Correlation of the 3-Gene TB Score at Baseline With Severity of Lung Pathology and Treatment Response in the Catalysis Treatment Response Cohort A, The 3-gene tuberculosis (TB) score at baseline (prior to treatment initiation) in patients with active TB (ATB) correlated with total glycolytic ratio activity (TGA) at baseline. Each dot indicates a patient. Black line indicates linear regression. Shaded region indicates 95% CI. B, The 3-gene TB score at day 168 (end of treatment [EOT]) in patients with ATB correlated with day 168 TGA. Each dot indicates a patient. Black line indicates linear regression. Shaded region indicates 95% CI. C, The 3-gene TB score at baseline for patients with clear radiology by 6 months (mean, −0.84; 95% CI, −0.73 to −0.95) was significantly lower than those with persistent lung inflammation (mean, −0.16; 95% CI, −0.05 to −0.27). Each dot represents a patient. Color scale reflects log_10_ day 168 Total Glycolytic Activity Index score. D, Patients with ATB with 3-gene TB scores above the median had significantly higher likelihood of prolonged lung pathology as measured by positron emission tomography–computed tomography.

The 3-gene TB score at diagnosis significantly correlated with lung pathology at diagnosis in patients with ATB as measured by TGAI using PET-CT (*r* = 0.54; *P* < .001) (Figure 4A). The 3-gene TB score at EOT also had a significant correlation with TGAI at EOT (*r* = 0.41; *P* < .001) (Figure 4B).

It has been previously reported that the presence of *Mtb* messenger RNA with nonresolving and intensifying lesions on PET-CT images indicated that many apparently cured patients may have subclinical ATB. A substantial proportion of patients with ATB in the CTRC showed PET-CT imaging response that was consistent with ATB after a 6-month treatment and 1-year follow-up.^15,21^ These patients were positive for *Mtb* RNA in sputum and bronchoalveolar lavage samples and included those with a durable cure and those who later developed recurrent disease. We defined the patients with ATB in the CTRC with a TGAI score of 400 or less at 6 months as radiologically clear, and those with a TGAI score higher than 400 at 6 months as having radiologically persistent lung inflammation. The 3-gene TB score at the time of diagnosis was significantly lower in the radiologically clear patients than those with persistent lung inflammation after 6 months of treatment (*P* < .001) (Figure 4C). Further, the 3-gene TB score at EOT continued to be higher in those with persistent lung inflammation compared with those who were radiologically clear at EOT (eFigure 2 in the Supplement).

Subsequently, we divided the patients with ATB in the CTRC cohort in 2 equal groups using the median of the 3-gene TB score at the time of diagnosis. We chose median instead of mean as it is robust to outliers. Patients with ATB with 3-gene TB score above the median had significantly higher likelihood of prolonged lung pathology as measured by PET-CT (hazard ratio, 3.41; 95% CI, 2.03-5.74; *P* < .001) (Figure 4D).

Collectively, across these 3 cohorts the 3-gene TB score had 99.3% NPV at 4% prevalence (eFigure 3 in the Supplement) and 70% specificity at 90% sensitivity for diagnosis of ATB (Table), meeting the WHO TPP for the non–sputum-based triage test. Further, the 3-gene TB score also identified individuals with LTBI that progressed to ATB 6 months prior and satisfied the WHO TPP for anticipating progression from LTBI to ATB with 18.3% PPV at 4% prevalence. Finally, the 3-gene TB score at the time of diagnosis was correlated with ongoing inflammation in lungs of patients with ATB as measured by PET-CT and was associated with patients who have persistent lung inflammation after the EOT.

## Discussion

We previously identified a blood-based 3-gene TB score that distinguishes patients with ATB from healthy controls and those with LTBI and other diseases independent of age, HIV coinfection, and prior BCG vaccination.^18^ The 3-gene TB score accurately diagnosed patients with ATB among heterogeneous populations in both active (BASS) and passive (ACS and CTRC) case findings. The score was significantly associated with the progression of individuals from LTBI to ATB 6 months prior to positive sputum test results. Finally, the 3-gene TB score at baseline correlated with the severity of lung inflammation in patients with ATB. The patients with a higher 3-gene TB score at the time of diagnosis had persistent lung inflammation at the EOT.

The 3-gene TB score satisfied the WHO TPP for a non–sputum-based triage test,^7^ demonstrating its potential for systemic screening to identify those who should be tested further for confirming diagnosis of ATB. In addition, the 3-gene TB score also satisfied the WHO TPP for a test anticipating progression from LTBI to ATB criteria^2^ with 18.3% PPV at 4% prevalence in the ACS cohort for identifying those with LTBI who will progress to ATB up to 6 months prior. The PPV of the 3-gene TB score to anticipate progression to ATB is more than 3-fold higher than required according to the FIND intervention TPP.

There is an unmet need for noninvasive or minimally invasive biomarkers that can be used in clinical trials and during treatment for ATB as a proxy of lung pathology. To our knowledge, the 3-gene TB score is the first example of applying a TB signature to PET-CT data from an independent study that had not been trained on PET-CT. The studies further observed that the 3-gene TB score at the time of diagnosis was significantly associated with 6-month radiological outcome. Patients with a 3-gene TB score higher than the median at time of diagnosis were 3 times more likely to have persistent lung inflammation.

The 3-gene TB score measured at the EOT was higher in those with ongoing lung inflammation, and accurately identified those who failed treatment as defined by a positive culture at the EOT. These results indicate that the 3-gene TB score may be useful in measuring lung pathology and monitoring treatment response because patients often cannot generate sputum after 6 months of treatment, and can be easily lost to follow-up when culture results take 40 to 60 days to declare negativity.

Since its publication in 2016,^18^ the 3-gene TB score is now shown to distinguish patients with ATB from those with LTBI, other diseases, and healthy controls in 19 independent cohorts consisting of over 3000 individuals from 14 countries across all age groups (children, adolescents, and adults) with or without HIV coinfection. To our knowledge, the 3-gene TB score is the first signature demonstrated to work in an active screen study, and to demonstrate that peripheral immune response can be a reasonable proxy for ongoing lung pathology during ATB. Collectively, these results show a substantial potential for the 3-gene TB score to monitor an individual with a latent *Mtb* infection through progression to and diagnosis of ATB and evaluate response to treatment.

### Limitations

There are several limitations of our analysis. First, the optimal threshold for diagnosis is platform dependent (eg, microarray, RNA-seq, and RT-qPCR). Two different technologies were used (RNA-seq and RT-qPCR) across 3 different cohorts. Because there is not yet a commercially available version of the test, we do not have a universal 3-gene TB score threshold for diagnosing ATB at the time. Such a threshold can only be determined once the 3-gene TB score is ported to a point-of-care device. Instead, we chose to report specificity corresponding with 90% sensitivity in each cohort with regard to the WHO TPP for a non–sputum-based triage test. Second, in the BASS, use of solid media instead of liquid culture and lack of radiography would arguably misclassify a number of patients. However, such a misclassification would likely bias performance of the 3-gene TB score toward the null hypothesis, leading to underestimate of its specificity. Third, only 7 of the individuals in the CTRC had failed treatment. Hence, further validation is required to better understand how closely the 3-gene TB score tracks with response to treatment. Fourth, the 3-gene TB score reliably identified progressors only up to 6 months prior to diagnosis, but not in samples beyond 6 months prior to diagnosis. It is currently not possible to say how long it takes for an individual with LTBI to progress to ATB. Fifth, arguably the correlation between the 3-gene TB score and TGAI measured in PET-CT was moderate. However, the 3-gene TB score at baseline also correlated with persistent PET-CT activity at the EOT, identifying patients with ATB who have subclinical TB. Therefore, although the 3-gene TB score moderately correlated with lung pathology, it provides additional clinically useful information that no other biomarker to date provides. Sixth, the 3-gene TB score satisfied the WHO TPP for a non-sputum-based triage test when we pooled results across the 3 cohorts. However, at 90% sensitivity there was a large variation in the specificity across the 3 cohorts. Hence, an active screen study with larger sample size is required to better estimate specificity. Despite these limitations, the results of our analyses are consistent with previously published results in more than 3000 samples.

## Conclusions

Across 3 independent cohorts in different clinical settings, the 3-gene TB score closely matches the WHO TPP benchmarks for a non–sputum-based triage test at high NPV. The 3-gene TB score is associated with disease progression, performs well in active case finding, and tracks with treatment response and lung pathology. These performance characteristics make it a potential test for ruling out ATB and a strong candidate for monitoring the status of an individual over the course of infection, disease, and treatment.

## Notes

Supplement. **eAppendix.** Supplemental Methods **eTable 1.** BASS Cohort Demographics **eTable 2.** Cohort Details **eFigure 1.** 3-gene TB Score in CTRC Before the Start of Treatment and at the End of Treatment **eFigure 2.** 3-gene TB Score at the End of Treatment Is Higher in Those With Persistent Lung Inflammation at the End of Treatment **eFigure 3.** Positive and Negative Predictive Value of 3-Gene TB Score at 90% Sensitivity and 70% Specificity **eReferences**
