# Supplementary material for: Assessment of Validity of a Blood-Based 3-Gene Signature Score for Progression and Diagnosis of Tuberculosis, Disease Severity, and Treatment Response
Source: JAMA Netw Open. 2018 Oct 26;1(6):e183779. doi: 10.1001/jamanetworkopen.2018.3779 (PMC6324428; doi:10.1001/jamanetworkopen.2018.3779)
Supplement: Supplement. — eAppendix. Supplemental Methods eTable 1. BASS Cohort Demographics eTable 2. Cohort Details eFigure 1. 3-gene TB Score in CTRC Before the Start of Treatment and at the End of Treatment eFigure 2. 3-gene TB Score at the End of Treatment Is Higher in Those With Persistent Lung Inflammation at the End of Treatment eFigure 3. Positive and Negative Predictive Value of 3-Gene TB Score at 90% Sensitivity and 70% Specificity eReferences [file jamanetwopen-1-e183779-s001.pdf]

# Supplementary Online Content

Warsinske HC, Rao AM, Moreira FMF, et al. Assessment of validity of a blood-based 3-gene signature score for progression and diagnosis of tuberculosis, disease severity, and treatment response. *JAMA Netw Open*. 2018;1(6):e183779. doi:10.1001/jamanetworkopen.2018.3779

**eAppendix.** Supplement Methods
**eTable 1.** BASS Cohort Demographics
**eTable 2.** Cohort Details
**eFigure 1.** 3-Gene TB Score in CTRC Before the Start of Treatment and at the End of Treatment
**eFigure 2.** 3-Gene TB Score at the End of Treatment Is Higher in Those With Persistent Lung Inflammation at the End of Treatment
**eFigure 3.** Positive and Negative Predictive Value of 3-Gene TB Score at 90% Sensitivity and 70% Specificity
**eReferences**

This supplementary material has been provided by the authors to give readers additional information about their work.

© 2018 Warsinske HC et al. *JAMA Network Open*.

**eAppendix.** Supplement Methods

## Cohort Descriptions

### *Adolescent Cohort Study (ACS)*

Patients in the ACS were diagnosed as LTBI by a positive QuantiFERON TB GOLD In-Tube Assay and/or a positive tuberculin skin test. Sputum and blood samples were collected from these adolescents every six months for 2 years and tested for development of ATB by sputum conversions. Out of 3,595 enrolled adolescents, whole blood samples from 153 LTBI adolescents were profiled using RNA-seq. As stated in the main manuscript, phenotypic information was only available for 144 individuals. Therefore, 7 adolescents were excluded from further analysis. Out of 144 individuals, 43 developed ATB during the study, called and are referred to as "progressors"; the remaining 101 individuals are referred to as "non-progressors." ATB diagnosis was determined by either two sputum smear tests positive for acid-fast bacilli or one *M. Tuberculosis* confirmed sputum culture (mycobacterial growth indicator tube, BD BioSciences).

### *Brazil Active Screening Study (BASS) Cohort*

The two prisons included in the BASS are approximately 200 kilometers from one another in the state of Mato Grosso do Sul, of similar size and demographics, with comparable access to medical services. Prior studies in these two prisons have demonstrated high burden of TB in both prisons and frequent transfer of inmates between the prisons. As site-specific differences in gene expression were unlikely in these nearby prisons in the same correctional system, the cohort was analyzed jointly.

Each participant in the BASS underwent an interview utilizing a standardized questionnaire. The variables obtained during the interview included sex, marital status, education, smoking history, illicit drug use, diabetes, contact with a TB case (in the household or elsewhere), the presence of a Bacillus Calmette-Guérin (BCG) vaccine scar (as determined by inspecting the participant's arm), previous incarceration, and TB symptoms (**eTable 1**). The participant's race/skin color (i.e., white, black, indigenous, Asian or mixed) was self-reported. In these prisons, our studies have found that 1.5% of inmates are HIV-infected[1,2]; we did not exclude HIV-infected individuals from the study, but happened to have none among the 81 participants.

Solid media culture was used because MGIT was not available at the state TB laboratory where the study was performed. Radiography was not available in the prisons, and a TB case was therefore defined as the presence of at least one positive culture test. Potential bias caused by the use of solid media culture is address in the limitations section of the main manuscript.

### *CTRC cohort*

In the CTRC, lesion morphology was characterized and measured based on CT images. PET uptake intensity of prominent lesions was measured using the mean and maximum standardized uptake value (SUV) and compared across time-points, using the uptake in the right lobe of the liver and the mediastinal aortic blood pool as reference values to account for variability in background uptake.

Sputum cultures were performed throughout the course of the study. Patients who completed therapy and had at least the last two consecutive sputum cultures at the end of treatment as negative were considered cured. Seven patients with positive sputum test at the end of treatment were considered to have failed treatment. Two patients with end of treatment (EOT) contaminated sputum cultures were considered unevaluable, and were removed from the analysis.

## Statistical Analysis

### *Receiver operator characteristic curves*

In the ACS, we used ROC curves to evaluate the ability of our signature to differentiate individuals that would eventually progress from LTBI to ATB from those that would not progress within different time windows . In the CTRC, we used ROC curves to evaluate the ability of the 3-gene TB score to differentiate those who succeeded in treatment from those who failed treatment at the end of treatment (day 168 for all individuals in the CTRC). We used the Hanley method to calculate AUROC and 95% confidence intervals[1,3,4].

### *Analysis of variance*

We used analysis of variance (ANOVA) to determine statistical difference in multiple-group comparisons and to evaluate the significance of time in these comparisons. In the ACS, we used ANOVA to compare the 3-gene TB score of progressors and non-progressors across time intervals. ANOVA was performed using the aov base function in R[5].

### *Mixed effects regression*

We used mixed effects regression to identify the effect of interactions between variables on the 3-gene TB score. In the ACS, we used mixed effects regression to determine if there is a significant effect of the interaction between time and progressor status on the 3-gene TB score. We also investigated if time, independent of disease status, had a significant effect of 3-gene TB score. Our model considered the 3-gene TB score to be a dependent variable with time and progressor status as fixed effects and the intercept by subject as a random effect. Linear mixed effects regression was performed using the lme package in R[6].

### *Pearson Correlations*

We used Pearson's method to calculate correlation and significance of the 3-gene TB score with other continuous phenotypic variables. In the CTRC we used Pearson correlation to determine the correlation between 3-gene TB score and TGAI from PET-CT at baseline and at the end of treatment. We calculated Pearson's correlations using the cor.test base function in R[5], which also computed p-values for statistical significance.

### *Student's t-test*

We used Student's t-test to determine statistical difference in two-group comparisons. p-value $\leq 0.05$ was considered significant and the t.test function in R was used to calculate significance[30]. In the CTRC, we used the student's t-test to compare the baseline 3-gene TB score between patients with ATB that have cleared lung inflammation at the end of treatment to those that had persistent lung inflammation at the end of treatment. We also

© 2018 Warsinske HC et al. *JAMA Network Open*.

compared the end of treatment 3-gene TB score between those groups using the Student's t-test.

***Cox hazard ratio***

We used Cox hazard ratio to determine significant hazard. P-value ≤ 0.05 was considered significant and the coxph and Surv functions from the Survival R package was used to calculate hazard ratio[35]. In the CTRC we used Cox hazard ratio to compare the likelihood of prolonged lung pathology given a high (above the median) baseline 3-gene TB score.

© 2018 Warsinske HC et al. *JAMA Network Open*.

**eTable 1.** BASS Cohort Demographics

| **Variables** | **Cases**<br>**n= 33** No. (%) | **Controls**<br>**n= 48** No. (%) | ***p*-value** |
|---|---|---|---|
| Age > 30 | 16 (48) | 17 (35) | 0.24 |
| Black or mixed race | 22 (67) | 34 (71) | 0.69 |
| Resided in Mato Grosso do Sul | 23 (70) | 35 (73) | 0.75 |
| Less than 8 years of schooling | 24 (73) | 23 (48) | 0.02 |
| Current cigarette smoker | 23 (70) | 28 (58) | 0.29 |
| Drug use during the last year | 12 (36) | 12 (25) | 0.27 |
| Alcohol use | 9 (27) | 20 (42) | 0.18 |
| Previous TB | 1 (3) | 0 (0) | 0.22 |
| HIV positive | 0 (0) | 0 (0) | >0.99 |
| Previously incarcerated | 31 (94) | 37 (77) | 0.04 |

© 2018 Warsinske HC et al. *JAMA Network Open*.

**eTable 2.** Cohort Details

| Cohort | No. of study participants | Patients with ATB | Patients with LTBI | Patients with other diseases | Healthy controls | Age range | ATB diagnosis criteria |
|---|---|---|---|---|---|---|---|
| **ACS** | 144 | 43 | 101 | 0 | 0 | 12-18 | positive sputum microscopy or culture |
| **BASS** | 81 | 33 | N/A* | N/A* | 48* | 20-72 | positive culture |
| **CRTC** | 138 | 100 | 0 | 17 | 21 | 17-67 | positive culture |

* BASS likely includes individuals with unknown LTBI and other diseases in its controls. These may not be healthy controls, but are listed as such for simplicity.

© 2018 Warsinske HC et al. *JAMA Network Open*.

**eFigure 1.** 3-Gene TB Score in CTRC Before the Start of Treatment and at the End of Treatment

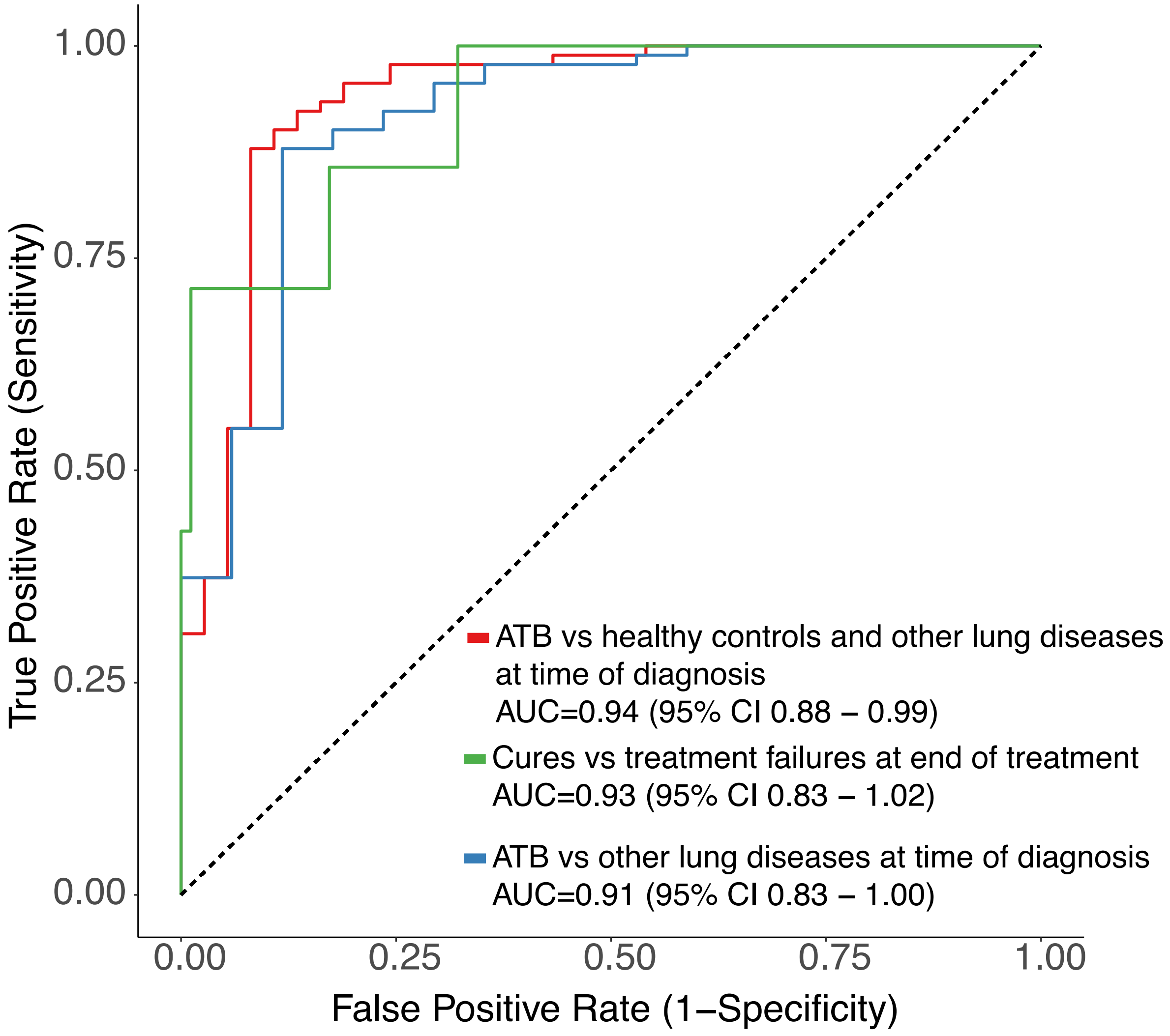


Red line depicts ROC distinguishing ATB from controls including. AUC = 0.94 (95%CI 0.88-0.99). Green line depicts ROC distinguishing patients with ATB that failed treatment by day 168 from those that cured by day 168. AUC = 0.93 (95% CI 0.83-1.02). Blue line depicts ROC distinguishing patients with ATB from patients with other diseases at time of diagnosis. AUC = 0.91 (95% CI 0.83-1.00).

© 2018 Warsinske HC et al. *JAMA Network Open*.

**eFigure 2.** 3-Gene TB Score at the End of Treatment Is Higher in Those With Persistent Lung Inflammation at the End of Treatment

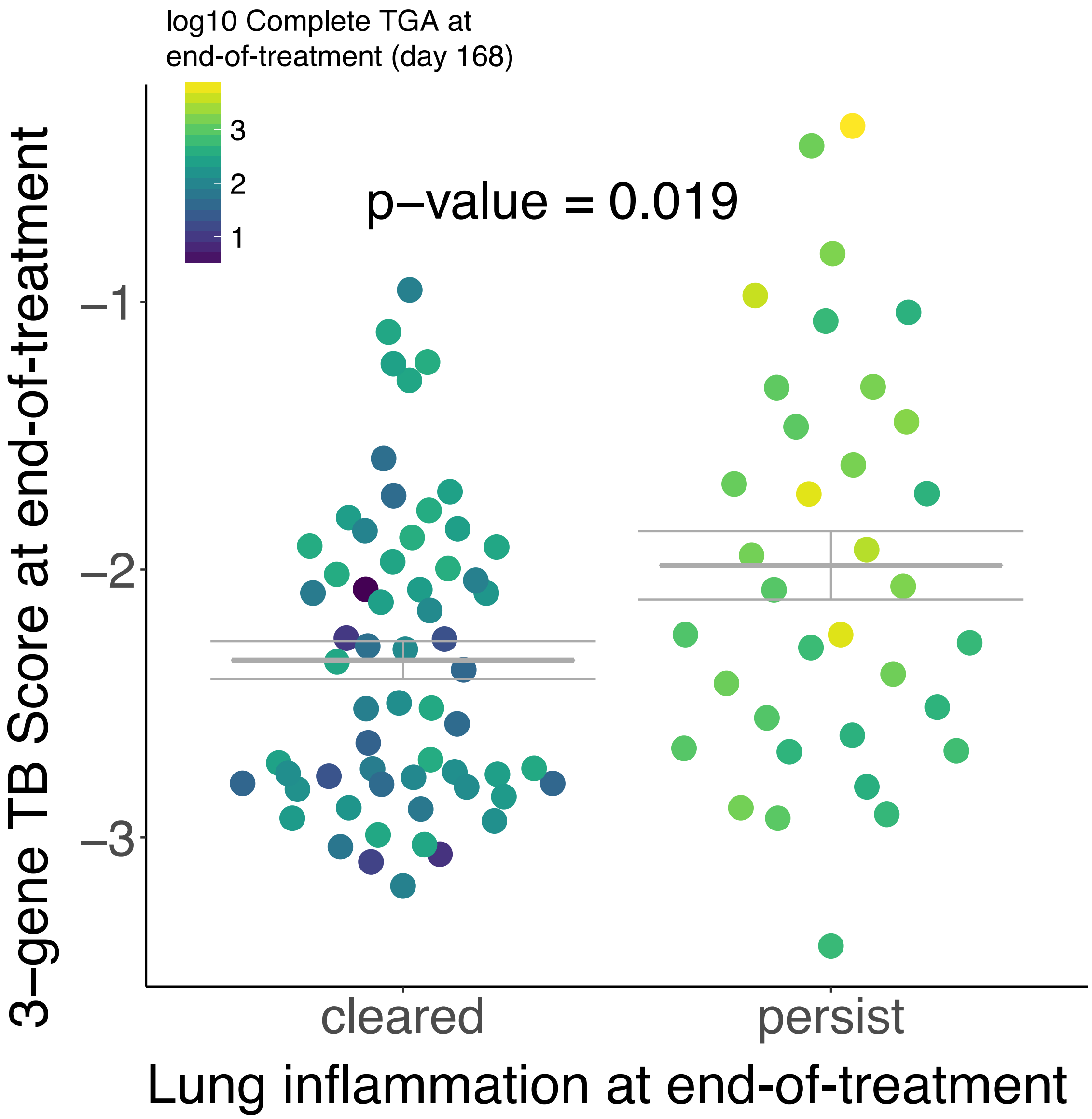


3-gene TB score at the end of treatment for patients with clear radiology by 6 months is significantly lower than those with persistent lung inflammation (p=0.019). Each dot represents a patient. Color scale reflects log 10 day 168 TGAI score.

© 2018 Warsinske HC et al. *JAMA Network Open*.

**eFigure 3.** Positive and Negative Predictive Value of 3-Gene TB Score at 90% Sensitivity and 70% Specificity

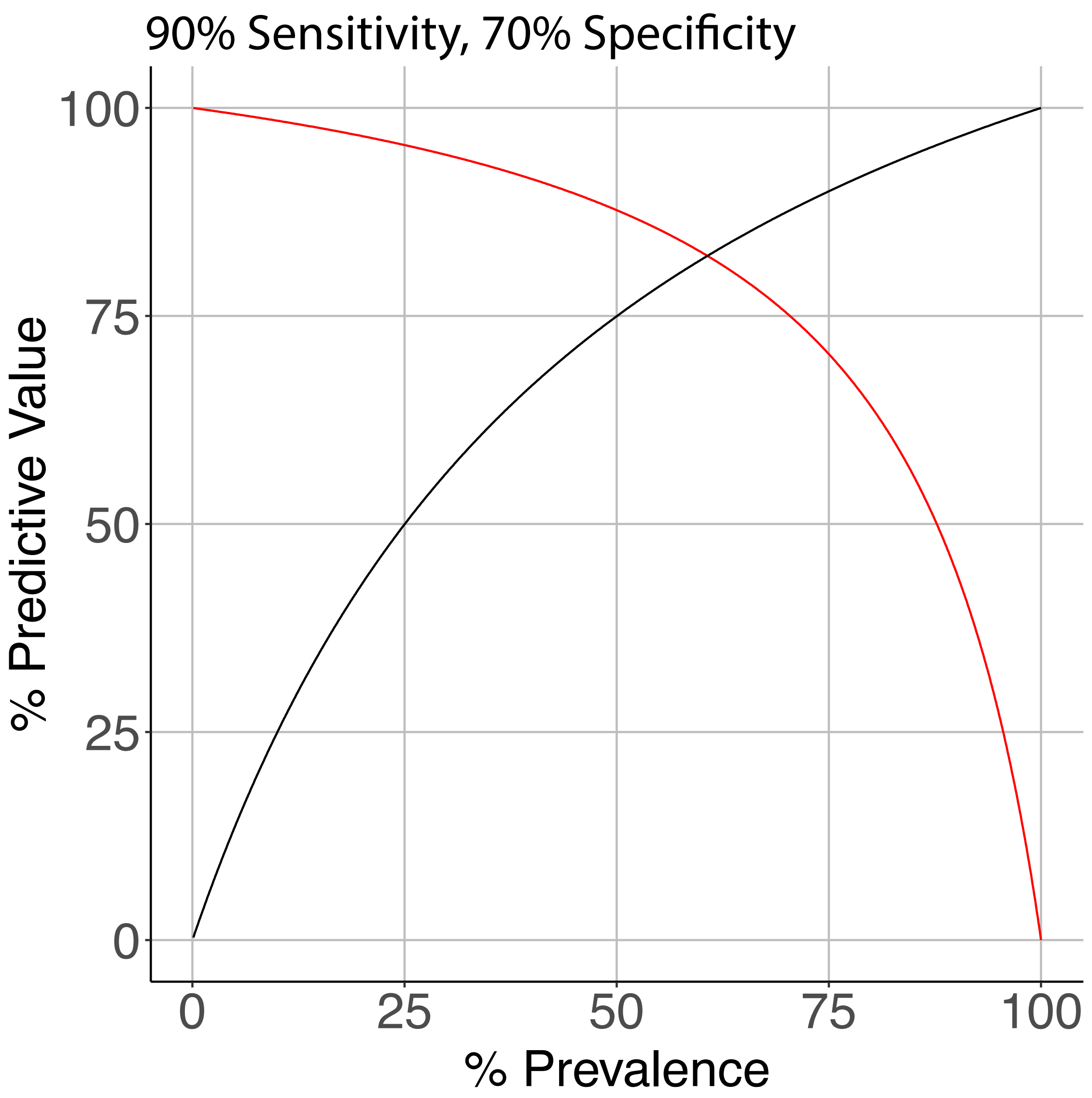


The red line depicts the negative predictive value (NPV) of the 3-gene TB score across prevalence. The black line depicts positive predictive value (PPV) of the 3-gene TB score across prevalence. At 90% sensitivity the 3-gene TB score Achieves a specificity of 70%.

© 2018 Warsinske HC et al. *JAMA Network Open*.

**eReferences**

**1.** Carbone AaS, Paião DS, Sgarbi RV, et al. Active and latent tuberculosis in Brazilian correctional facilities: a cross-sectional study. *BMC Infect Dis.* 2015;15:24.

**2.** Sgarbi RV, Carbone AaS, Paião DS, et al. A Cross-Sectional Survey of HIV Testing and Prevalence in Twelve Brazilian Correctional Facilities. *PLoS One.* 2015;10(10):e0139487.

**3.** Altman DG, Bland JM. Diagnostic tests 2: Predictive values. *BMJ.* 1994;309(6947):102.

**4.** Bayes, M., Price M. *An Essay towards Solving a Problem in the Doctrine of Chances.* Philosophical Transactions of the Royal Society of London1763.

**5.** Team RC. R: A Language and Environment for Statistical Computing. In: Computing RFfS, ed. Vienna, Austria: https://www.R-project.org/; 2016.

**6.** Bates D, Maechler M, Bolker B, Walker S. Fitting Linear Mixed-Effects Models Using {lme4}. *Journal of Statistical Software.* 2015.

© 2018 Warsinske HC et al. *JAMA Network Open*.
